# Linking the phenotype of *SNCA* Triplication with PET-MRI imaging pattern and alpha-synuclein CSF seeding

**DOI:** 10.1038/s41531-022-00379-8

**Published:** 2022-09-15

**Authors:** Isabel Wurster, Corinne Quadalti, Marcello Rossi, Ann-Kathrin Hauser, Christian Deuschle, Claudia Schulte, Katharina Waniek, Ingolf Lachmann, Christian la Fougere, Kathrin Doppler, Thomas Gasser, Benjamin Bender, Piero Parchi, Kathrin Brockmann

**Affiliations:** 1 Center of Neurology, Department of Neurodegeneration and Hertie-Institute for Clinical Brain Research, University of Tuebingen, Tuebingen, Germany; 2 German Center for Neurodegenerative Diseases, University of Tuebingen, Tuebingen, Germany; 3 IRCCS, Istituto Delle Scienze Neurologiche di Bologna, Bologna, Italy; 4 Department of Experimental, Diagnostic and Specialty Medicine (DIMES), University of Bologna, Bologna, Italy; 5 Roboscreen GmbH, Leipzig, Germany; 6 Department of Nuclear Medicine and Clinical Molecular Imaging, University of Tuebingen, Tuebingen, Germany; 7 Department of Neurology, University Hospital of Wuerzburg, Wuerzburg, Germany; 8 Center of Neuroradiology, University of Tuebingen, Tuebingen, Germany

**Keywords:** Parkinson's disease, Diagnostic markers

## Abstract

Lewy-body pathology with aggregation of abnormal conformations of the protein alpha-synuclein (α-Syn) represent the histopathological hallmarks of Parkinson’s disease (PD). Genetic prototypes such as PD due to mutations in the *alpha-synuclein* gene (*SNCA*) offer the opportunity to evaluate α-Syn-related profiles in patient-derived biomaterial. We identified a family with a *SNCA* triplication and assessed the index patient for CSF α-Syn seeding capacity and levels of total α-Syn along with other neurodegenerative CSF markers (Aβ_1-42_, total-Tau, phospho-Tau, NFL). As no published CSF data in patients with *SNCA* triplication are available, we descriptively compared his CSF profiles to those of sporadic PD patients and PD patients with *GBA* mutations as these are also specifically associated with prominent α-Syn pathology. Additionally, skin biopsies with staining for phospho-α-Syn were done. To assess cerebral glucose metabolism and brain atrophy combined positron emission tomography and magnetic resonance imaging ([^18^F]FDG-PET/MRI) was performed. Age at onset was 24 years and motor impairment was accompanied by prominent non-motor symptoms with early development of dementia, depression, REM sleep behavior disorder, hyposmia, and dysautonomia. Correspondingly, PET-MRI showed hypometabolism and atrophy in frontal, temporoparietal and occipital regions. CSF levels of total α-Syn were threefold higher and RT-QuIC showed remarkable α-Syn seeding activity in all kinetic categories in the SNCA_Triplication_ patient compared to patients with *GBA* mutations. Our results are consistent with findings that not only mutant forms but also overexpression of the wild-type α-Syn protein lead to PD and PD dementia and show a striking CSF α-Syn seeding profile, thus substantiating the role of RT-QuIC as a specific in vivo biomarker of α-Syn brain pathology.

## Introduction

Genetic and sporadic forms of PD share many overlapping features. Next to parkinsonism due to nigrostriatal dopaminergic degeneration, Lewy-body pathology with aggregation of abnormal conformations including posttranslational modifications of the protein alpha-synuclein (α-Syn) represent the histopathological hallmarks. Thus, findings in genetic forms help to identify common pathogenic mechanisms and to understand the specific molecular pathways underlying PD^1^.

Point mutations along with duplications and triplications of the wild-type *alpha-synuclein* gene (*SNCA*) cause autosomal dominantly inherited PD^2–5^. Patients affected by *SNCA* point mutations present a clinical phenotype similar to those with sporadic PD. However, they have an earlier age at onset and often suffer from dementia and autonomic disturbances^6^. Since patients with *SNCA*-triplications (SNCA_Triplication_) present with an early age at onset, rapid disease progression, prominent dementia, and frequent dysautonomia while patients with *SNCA*-duplications show a more typical late-onset PD-phenotype, severity of the clinical trajectories in *SNCA* multiplication seems to be associated with gene dosage^5^. In line with these clinical findings, neuropathological studies in patients with *SNCA* multiplication show diffuse Lewy body pathology similar to that seen in dementia with Lewy bodies (DLB)^7,8^. These characteristics are of major importance as they demonstrate that both, toxic-gain of function of mutant protein as well as overproduction of the wild-type protein lead to aggregate formation.

With disease-modifying compounds such as monoclonal antibodies or active vaccination targeting α-Syn currently tested in clinical trials, patient stratification according to α-Syn-specific enrichment strategies is a much-needed prerequisite. Clearly defined genetic forms such as *SNCA*-associated PD offer the opportunity to validate findings from experimental studies in cell and animal models in patient-derived biomaterial and evaluate the validity of assays assessing α-Syn. As there are no reliable imaging marker to assess the cerebral load of α-Syn in vivo, research has focused on CSF. Yet, it is unclear whether CSF levels of α-Syn species reflect α-Syn brain pathology. Recently, highly sensitive seed amplification assays (SAA) such as the real‐time quaking‐induced conversion (RT‐QuIC) and protein misfolding cyclic amplification (PMCA) have been successfully implemented. These assays exploit the seeding capacities of prion and prion-like proteins using an amplification strategy to reveal minute amounts of disease-specific protein aggregates in CSF^9,10^. Both methods show a high sensitivity (88–96%) and specificity (83–98%) for sporadic PD and DLB compared to controls^11^. Using RT-QuIC, we could recently show that both, PD and DLB patients with severe mutations in the gene *glucocerebrosidase* (*GBA*) present with the highest α-Syn seeding activity compared to other genetic forms with known variable (*LRRK2*) or even sparse Lewy body pathology (*Parkin*, *PINK1*)^12^. Thereby, these seeding assays might serve as a specific in vivo biomarker of α-Syn brain pathology. In this line of reasoning, we assessed an early-onset male patient with *SNCA* triplication for α-Syn seeding capacity and levels of total α-Syn in CSF along with other neurodegenerative-associated CSF levels of Aβ_1-42_, total-Tau, phospho-Tau, NFL. As no published CSF data in patients with *SNCA* triplication are available, we descriptively compared his CSF profiles to those of PD patients with *GBA* mutations (PD_GBA_) as these are also specifically associated with prominent α-Syn pathology^13^. Additionally, skin biopsies with staining for phospho-α-Syn were done. To assess cerebral metabolism and brain atrophy combined positron emission tomography and magnetic resonance imaging (PET-MRI) was performed.

## Results

### Demographic and clinical characteristics

The family is of European ancestry. The male SNCA_Triplication_ patient manifested with predominantly right-sided hypobradykinesia, rigidity, and resting tremor at the age of 24 years. At that time, [^123^I]FP-CIT (DaTScan®) single photon emission computed tomography (SPECT) showed a left accentuated striatal dopamine transporter (DAT) deficit. MRI was normal without evidence for secondary parkinsonism. Disease duration was 2 years and age was 26 years when we first saw him in our clinic. At that time, he presented with moderate bilateral hypobradykinesia, marked rigidity and resting tremor with predominance of the right side. In addition, frontal disinhibition (positive palmomental reflex, persisting glabella reflex) as well as ideatoric and ideomotor apraxia was seen along with prominent cognitive deficits (executive and amnestic impairment; MoCA 15 points), depression (BDI-II 27 points), REM sleep behavior disorder (RBD), daytime sleepiness, hyposmia and orthostatic dysfunction. No compulsive or impulse control behavioral disorder or hallucinations were reported. Levodopa equivalent daily dosage (LEDD) was 600 mg. After these 2 years of disease duration, he already manifested with motor and non-motor fluctuations with levodopa-induced dyskinesia in the ON state and severe bradyphrenia, apathy and depression during wearing-OFF and OFF state.

**Fig. 1:**
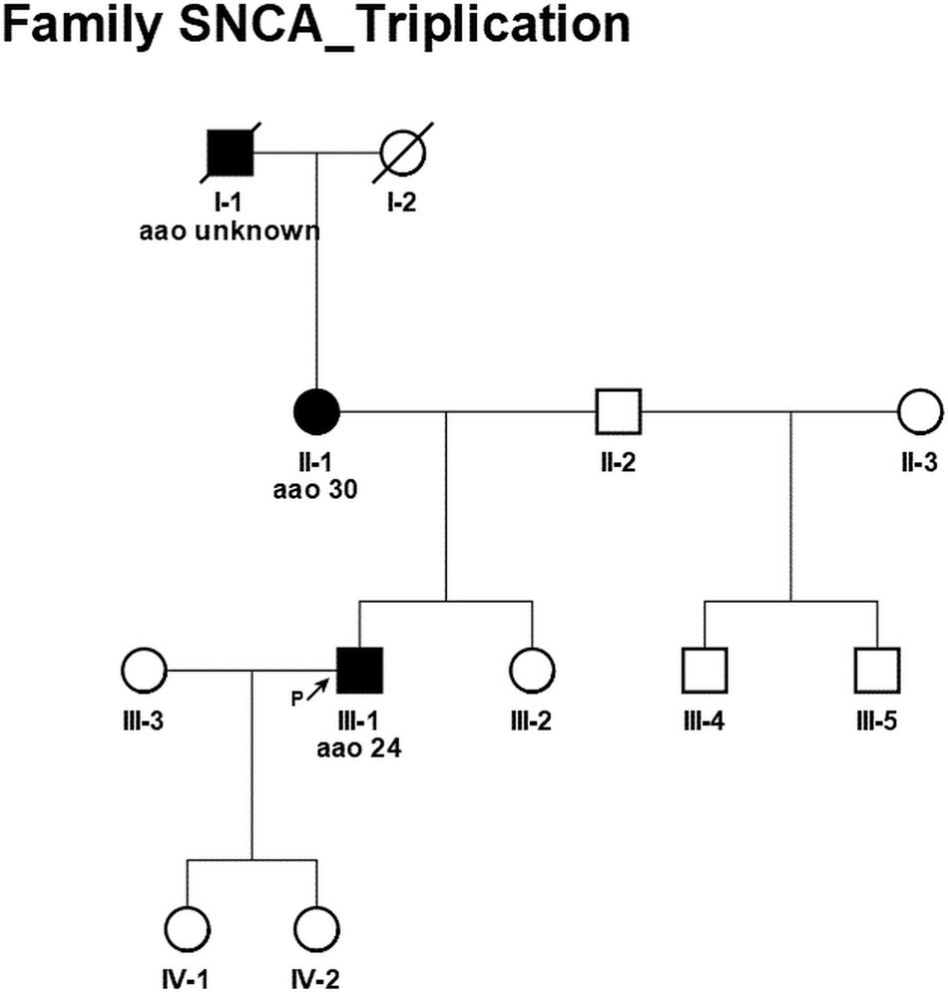
Pedigree of the *SNCA* Triplication family.

The family history was positive for PD and compatible with an autosomal dominant mode of inheritance. His mother manifested with PD by the age of 30 and currently, at the age of 50, suffers from severe PD dementia and lives bed-ridden in a nursing home. His grandfather in the mother’s line also suffered from PD. However, no clinical information could be obtained. The patient has one younger sister from the same mother who was reported to show no motor or non-motor symptoms and two half-brothers from another mother (one younger, one older), Fig. 1.

### PET-MRI

**Fig. 2:**
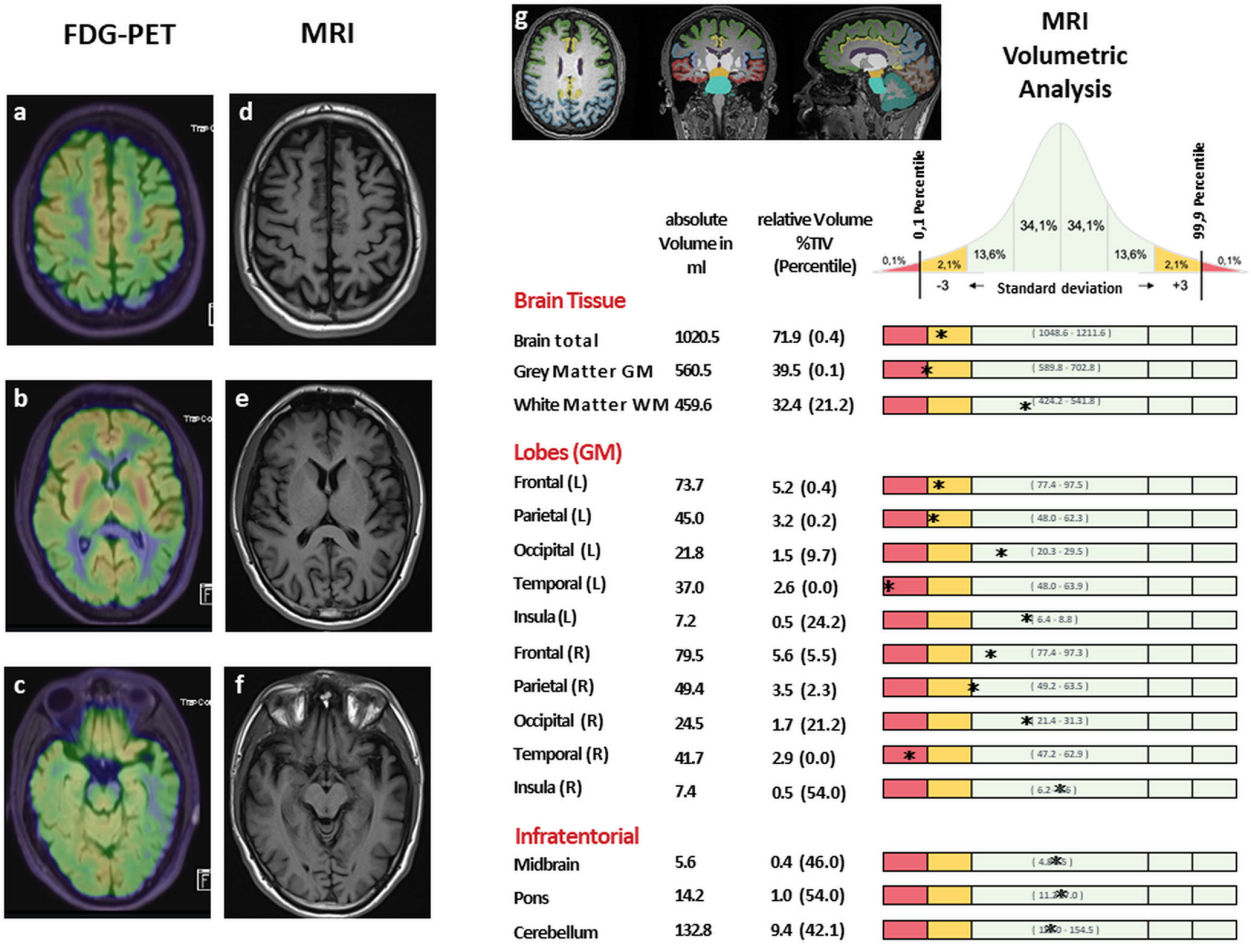
PET-MRI in the SNCA_Triplication_ Patient. [^18^F]FDG-PET showed bilateral hypometabolism in frontal (**a**), temporoparietal (**a** = parietal, **c** = temporal), and occipital (**b**) regions as well as of the precuneus and gyrus cinguli posterior. Contrary, the basal ganglia showed an increased metabolism (**b**). Corresponding to the functional hypometabolic pattern in FDG-PET, MRI showed predominantly left-sided frontal (**d**) and temporoparietal (**d** = parietal, **f** = temporal) atrophy while the basal ganglia appeared normal (**e**). Automated brain tissue and lobe segmentation with volumetric analysis using the AIRAmed software (**g**). * indicates the SNCA_Triplicatio__n_ patient's individual brain volume pattern corrected for total intracranial volume and compared (plotted as standard deviation) to an age and sex matched healthy control group that consists of over 8000 healthy brains from 18 to 85 years.

[^18^F]FDG-PET images revealed a bilateral reduced rCGM in part of the frontal, temporoparietal, and occipital cortex as well as in the precuneus and the posterior gyrus cinguli. In contrast, the basal ganglia showed an increased rCGM. Corresponding to the the glucose hypometabolism shown in the PET scan, visual as well as automated MRI analysis showed predominantly left-sided frontal and temporoparietal atrophy 3–4 standard deviations below age- and sex-matched reference brains with sparing of the hippocampus. With the involvement of regions in the temporal and occipital cortex, the overall pattern observed in PET/MRI was compatible with a diagnosis of PDD or DLB, Fig. 2.

### CSF α-Syn measurements

CSF levels of total α-Syn were threefold increased in the SNCA_Triplication_ patient (1792 pg/ml) compared to the PD_GBA_severe_ (PD_GBA_severe_ mean 512 pg/ml) and PD_wildtype_ (mean 578 pg/ml) groups.

**Fig. 3:**
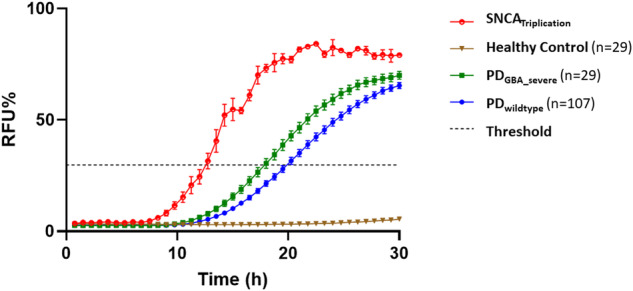
RT-QuIC positive replicates relative fluorescence curves at 30 h. RT-QuIC kinetics measured by the mean relative fluorescence (RFU) of positive curves were most prominent in the SNCA_Triplication_ patient when compared to PD_GBA_severe_ and PD_wildtype_. He showed the shortest time required to reach the threshold (LAG phase), highest area under the curve (AUC)and the peak of the fluorescence response (Imax). Each curve represents the average of the group, error bars indicate standard deviation, and the black dashed line indicates the threshold for positive seeding. Relative fluorescence unit (RFU) values are normalized to percentage against the maximum intensity of fluorescence of the respective experimental plate.

RT-QuIC α-Syn seeding was positive in all 4 replicates in the SNCA_Triplication_ patient. Moreover, RT-QuIC kinetics were most prominent in the SNCA_Triplication_ patient when compared to PD_GBA_severe_ and PD_wildtype_. He showed the shortest LAG phase (SNCA_Triplication_ 13 h, PD_GBA_severe_ mean 19 h, PD_wildtype_ mean 21 h). Correspondingly, AUC and Imax were highest in the SNCA_Triplication_ patient (AUC: SNCA_Triplication_ 1352, PD_GBA_severe_ mean 858, PD_wildtype_ mean 713; Imax: SNCA_Triplication_ 88, PD_GBA_severe_ mean 71, PD_wildtype_ mean 68), Table 1 and Fig. 3.

**Table 1:** RT-QuIC seeding profiles in PD stratified by mutation status.

	Controls *n* = 26	PD_wildtype_ *n* = 107	PD_GBA_risk_ *n* = 53	PD_GBA_mild_ *n* = 17	PD_GBA_severe_ *n* = 29	SNCA_Triplication_ *n* = 1
Male sex, %	54	70	70	65	66	100
Age (years)	59 ± 12	65 ± 8	65 ± 9	66 ± 9	59 ± 10	26
Age at onset (years)	–	61 ± 8	58 ± 10	57 ± 9	51 ± 10	24
Disease duration (years)	–	5 ± 3	8 ± 5	8 ± 6	9 ± 7	2
UPDRS III	2 ± 2	23 ± 10	28 ± 11	28 ± 13	27 ± 13	30
MoCA	27 ± 3	26 ± 3	24 ± 5	25 ± 6	24 ± 5	15
LEDD	–	389 ± 259	595 ± 363	626 ± 252	701 ± 545	600
RT-QuIC positive (%)	2 (8)	97 (91)	48 (91)	11 (65)	27 (93)	1 (100)
RT-QuIC 0/4 positive (%)	18 (92)	10 (9)	5 (9)	6 (35)	2 (7)	0 (0)
RT-QuIC 2/4 positive (%)	1 (4)	13 (12)	1 (2)	1 (6)	0 (0)	0 (0)
RT-QuIC 3/4 positive (%)	0 (0)	29 (27)	11 (21)	3 (18)	5 (17)	0 (0)
RT-QuIC 4/4 positive (%)	1 (4)	55 (52)	36 (68)	7 (41)	22 (76)	1 (100)
RT-QuIC AUC	–	713 ± 238	795 ± 281	797 ± 157	858 ± 179	1352
RT-QuIC Imax	–	68 ± 14	71 ± 15	72 ± 12	71 ± 10	88
RT-QuIC LAG	–	21 ± 3	20 ± 3	20 ± 2	19 ± 2	13
CSF total α-Syn, pg/ml	583 ± 181	578 ± 279	560 ± 212	489 ± 220	512 ± 273	1792
CSF Aβ_1-42_, pg/ml	925 ± 231	679 ± 249	684 ± 268	706 ± 214	779 ± 248	759
CSF t-Tau, pg/ml	240 ± 97	232 ± 127	265 ± 152	241 ± 100	203 ± 86	576
CSF p-Tau, pg/ml	41 ± 13	41 ± 16	39 ± 12	43 ± 15	36 ± 16	49
NFL, pg/ml	542 ± 239	894 ± 633	1042 ± 1320	1099 ± 965	839 ± 635	1212
Skin biopsy and RT-QuIC available			*n* = 3	*n* = 5	*n* = 1	*n* = 1
Positive phospho-α-Syn nerve fibers in skin biopsy/positive RT-QuIC α-Syn seeding			3/3	2/4	0/1	1/1

*MoCA* Montreal cognitive assessment, *UPDRS III* Unified Parkinson Disease Rating Scale part III, *LEDD* Levodopa equivalent daily dosage.

### CSF Aβ1-42, total-Tau, phospho-Tau, NFL

The classical CSF markers for Alzheimer’s disease Aβ_1-42_ and p181-Tau were within the clinically normal range (age-related cut-off: Aβ_1-42_ < 600 pg/ml; p181-Tau >57) in the SNCA_Triplication_ patient and comparable to PD_GBA_severe_ and PD_wildtype_. However, CSF levels of t-Tau and NFL as general markers for neuronal–axonal damage were above the age-related cut-off (t-Tau cut-off: >404, SNCA_Triplication_ = 576; NFL cut-off: >362, SNCA_Triplication_ = 1212) and also highest in the SNCA_Triplication_ patient compared to PD_GBA_severe_ and PD_wildtype_, Table 1.

### Skin biopsy phospho-α-Syn

**Fig. 4:**
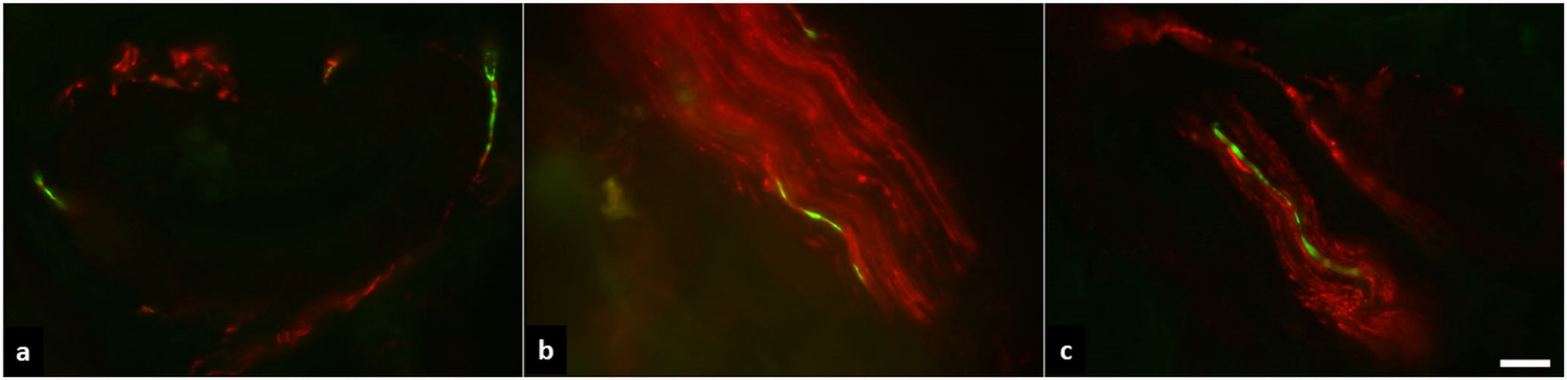
Phospho-α-Syn deposition in dermal biopsy. Representative photomicrographs of a double-immunofluorescence staining with anti-phospho-α-Syn (green) and anti-PGP9.5 (red), Scale bar = 20 µm. Phospho-α-Syn-positive nerve fibers were found in all three, autonomic vasomotor fibers (**a**), dermal nerve bundles (**b**), and pilomotor fibers (**c**) in both biopsies that were taken, from the distal and proximal leg.

In the SNCA_Triplication_ patient, phospho-α-Syn-positive nerve fibers were found in all three, dermal nerve bundles, autonomic vasomotor fibers, and pilomotor fibers in both biopsy sites that were taken, distal and proximal leg, Fig. 4. Only five out of nine PD_GBA_ patients showed positive phospho-α-Syn staining in skin biopsies, Table 1.

## Discussion

Albeit its rarity, the identification of this SNCA_Triplication_ family is of greatest value for α-Syn-related research in general for the following reasons: (I) It is consistent with findings that not only mutant forms but also overexpression of the wild-type α-Syn protein lead to PD and PD dementia. (II) It offers the opportunity to study α-Syn-specific profiles in patient-derived biomaterials and evaluate the validity of assays assessing alpha-synuclein in vitro such as the recently implemented RT-QuIC assay in a biologically outstanding role-model of α-Syn pathology. (III) It allows to assess cerebral patterns of metabolism and atrophy associated with distinct α-Syn pathology and clinical characteristics.

So far, nine families with SNCA_Triplication_ world-wide have been described in the literature. Clinical characteristics in our family with early onset of PD between 20 and 30 years of age accompanied by prominent non-motor symptoms with early development of dementia, depression, REM sleep behavior disorder, hyposmia and dysautonomia are in line with findings from the other SNCA_Triplication_ families^14^.

Corresponding to the multisystemic non-motor clinical symptoms with hyposmia, apraxia, dementia, RBD, and dysautonomia, [^18^F]FDG-PET/MRI showed marked cortical involvement with frontal, temporoparietal and occipital hypometabolism and atrophy. Similar [^18^F]FDG -PET and MRI patterns with fronto-temporo-parietal hypometabolism have been previously described in an Asian and in an Italian SNCA_Triplication_ patient highlighting the widespread cortical neurodegeneration early on in the disease course^15–17^.

Post-mortem studies in SNCA_Triplication_ patients showed severe neuronal degeneration of the substantia nigra and locus coeruleus along with widespread Lewy-body pathology in cortical, hippocampal, and hypothalamus regions as well as in basal nucleus of Meynert compatible with diffuse Lewy body disease^8^. Studies on expression levels in blood, post-mortem brain tissue, IPS-cell models and midbrain organoids from SNCA_Triplication_ patients clearly demonstrate that the levels of α-Syn are doubled and that this pure overload of the wildtype protein is sufficient to promote excessive protein aggregation^8,18–23^. One plausible explanation is that protein aggregation is a concentration-dependent phenomenon with insufficient clearance leading to oxidative stress and compromised autophagy with protein aggregation as shown in IPS cell models from SNCA_Triplication_ patients^21,22^.

Bridging the gap between clinical and imaging characteristics with in vivo biomarkers that validly reflect the underlying pathology is the ultimate goal for designing clinical trials. The missing piece in our SNCA_Triplication_ patient was to evaluate whether (in accordance with the aforementioned expression level studies in brain and cell models) levels of α-Syn are the driving force for the diffuse neurodegeneration seen in PET-MRI that led to such a complex and fast progressing phenotype. Indeed, CSF levels of total α-Syn were 3-fold higher than normal. This might seem somewhat odd as cross-sectional and longitudinal analyses in PD_GBA_wildtype_ and PD_GBA_ demonstrated decreased CSF levels of total α-Syn compared to healthy controls with the highest decrease in PD_GBA_ patients carrying severe variants^24–27^. Correspondingly, the same pattern was also reported in patients with DLB_GBA_^28^. It is hypothesized that due to impaired α-Syn clearance promoting its aggregation in the brain, CSF levels of total α-Syn become somewhat lower, similar to Aβ_1-42_ in Alzheimer’s disease. Of course, this is a rather simplistic way and the underlying mechanisms are more complex. We can only speculate why the SNCA_Triplication_ patient shows such high CSF levels of total α-Syn. The most reasonable explanation might be the extremely high expression of wildtype α-Syn due to the triplication. This might not only overwhelm the whole clearance system in a rather short time leading to protein aggregation (which according to the above-mentioned hypothesis would result in decreased levels of total α-Syn) but still produces so much wildtype protein represented by such high levels in CSF. In this context, it would be extremely valuable to assess patients with *SNCA* duplications as CSF levels of total α-Syn should be somewhat lower but still higher than in wildtype patients. Besides, the assessment of RT-QuIC seeding profiles would be also of interest. RT-QuIC showed remarkable CSF α-Syn seeding activity in all kinetic categories in the SNCA_Triplication_ patient compared to PD_GBA_severe_ and PD_wildtype_ while classical Alzheimer’s disease CSF profiles of Aβ_1-42_ and p181-Tau were normal. We could previously show that PD_GBA_severe_ present with the highest CSF α-Syn seeding activity compared to PD_wildtype_ and other genetic forms with known variable (*LRRK2*) or even sparse Lewy-body pathology (*Parkin*, *PINK1*)^12^. Adding this even more striking CSF α-Syn seeding profile of the SNCA_Triplication_ patient further highlights the role of RT-QuIC as specific in vivo biomarker of α-Syn brain pathology. One might speculate whether specific RT-QuIC kinetic parameters such as the LAG phase (time required to reach the threshold) are quantitatively indicative of the cerebral α-Syn load. Unfortunately, we as well as the PPMI study do not have other PD patients with SNCA mutations of whom CSF is available. Specifically, CSF profiles of the affected mother of the patient would be extremely valuable due to the same genetic background without further confounders. We tried to visit the mother in the nursing home but could not enter during the SARS-CoV2 pandemic. However, with efforts of the MJFF-funded projects “Global Genetic Consortium” and “GP2” additional cases with SNCA mutations will hopefully be identified.

Given the pronounced clinical, imaging and biomarker characteristics after two years of disease duration one might raise the academic discussion of a diagnosis of PD with dementia or DLB. Until now, our patient does not present with visual hallucination, a key feature for DLB. Moreover, according to the revised consensus criteria for DLB (McKeith), DLB should be diagnosed if dementia occurs before or concurrently with parkinsonism. This was not the case as the PD-associated motor manifestation clearly started before the cognitive decline. But of course, trajectories between PD–PDD–DLB represent a clinical and histopathological continuum and such cases raise the discussion on our definition, specifically with focus on a clinical or pathology-associated definition. This will be important for designing clinical trials.

This case further confirms that α-Syn pathology is not only restricted to the brain but also evident in the periphery in dermal nerve bundles, autonomic vasomotor fibers, and pilomotor fibers. However, while eight out of nine PD_GBA_ showed CSF RT-QuIC α-Syn-seeding, only five out of these eight showed positive phospho-α-Syn staining in skin biopsies. This indicates, that CSF RT-QuIC aSyn-seeding might be more sensitive for brain-related α-Syn aggregation.

In conclusion, we demonstrate how genetic triplication of the *SNCA* gene relates to a distinct α-Syn specific CSF biomarker profile, which in turn is associated with diffuse cerebral hypometabolism in ([^18^F]FDG-PET/MRI and a fast progressing clinical phenotype.

## Materials and methods

### Participants

#### SNCA family

The early-onset male index patient was seen in our outpatient clinic for Parkinsonian disorders in June 2021 for the first time. He reported a positive family history compatible with an autosomal-dominant mode of inheritance with his mother and grandfather (mother’s line) also affected by PD with PD-associated dementia (PDD). Consequently, genetic testing was initiated according to the German law for Genetic Diagnostics.

#### PD_wildtype_ and PD_GBA_ cohort

Between 2005 and 2020, ~2500 PD patients have been recruited from the outpatient clinic and/or ward for Parkinson’s disease at the University Hospital of Tuebingen. Of those, 497 PD patients agreed to lumbar puncture. Spouses of patients and volunteers recruited by newspaper advertisements were assessed to have no neurodegenerative disease and served as control participants. CSF samples of 107 randomly selected PD patients without mutation in *GBA* or *LRRK2* (PD_wildtype_), 99 PD_GBA_, and 26 healthy controls were analyzed for CSF levels of total α-Syn, α-Syn seeding capacity, Aβ_1-42_, total-Tau, phospho-Tau, NFL and reported previously^12^.

### Genetic analysis

#### SNCA triplication

Due to the early-onset manifestation in the male patient along with a family history suggestive of an autosomal-dominant mode of inheritance, a classical targeted gene-panel analysis comprising the PD-associated genes *GBA*, *LRRK2*, *SNCA*, *VPS35*, *PRKN*, *PINK1*, *DJ1*, *UCHL1*, *ATP13A2*, *FBXO7*, and *SLC6A3* was done. Since no mutation was identified, additional MLPA analysis of *SNCA*, *PRKN*, *PINK1*, and *DJ1* was performed. Thereby, four gene copies of *SNCA* were identified and further confirmed by qPCR.

#### GBA cohort

Genetic screening for mutations in the *GBA* gene was done as previously described^26^. According to established convention, all *GBA* variants were named based on the processed protein excluding the 39-residue signal peptide. *GBA*-subgroup classification of variant severity was done according to established genotype risks reported for PD: low risk (PD_GBA_risk_), mild risk (PD_GBA_mild_), severe risk (PD_GBA_severe_)^29^. All participants were controlled to have no p.G2019S *LRRK2* mutation.

### Clinical investigations

All participants were examined by a movement disorders specialist. Diagnosis of PD was defined according to UK Brain Bank Society Criteria^30^. Patients were assessed in dopaminergic ON state. Severity of motor symptoms was assessed using part III of the Unified Parkinson’s disease Rating Scale (UPDRS-III), from 2006-2008 the old version, from 2009 on the MDS-UPDRS^31^. Disease stage was categorized by the modified Hoehn and Yahr Scale (H&Y)^32^. Cognitive function was tested using the Montreal Cognitive Assessment (MoCA) and/or Mini Mental Status Examination (MMSE)^33^. Since the MoCA was available only from 2009 on, previously obtained MMSE scores were converted into MoCA equivalents^34^.

### [^18^F]FDG-PET/MRI

To assess regional cerebral glucose-metabolism (rCGM) and morphological changes (e.g. brain atrophy) combined Positron-Emission-Tomography and 3Tesla Magnetic-Resonance-Imaging (PET/MRI) was performed (mMR, Siemens Healthineers, Erlangen, Germany). PET-data were acquired 1 h after intravenous injection of 181 MBq [^18^F]FDG simultaneously to multiparametric MRI. Reconstructed, attenuation and scatter corrected PET-images were evaluated with a dedicated software (Brass®, Hermes Medical Solutions, Sweden). Next to standard neuroradiological MRI evaluation, an unbiased quantitative volumetric analysis was performed using the AIRAmed (Artificial Intelligence in RAdiology) software. For this procedure, an MPRAGE sequence with 1.0 × 1.0 × 1.0 mm^3^, TR: 2300 ms, TE: 3.37 ms, TI: 900 ms, FA: 9 was used. By this algorithm brain tissue is segmented into gray and white matter. Gray matter is further segmented anatomically into the different lobes (frontal, temporal, parietal, occipital, limbic) and infratentorial structures (cerebellum, midbrain, pons). The patient’s individual volumetric pattern was corrected for total intracranial volume and compared to an age and sex matched healthy control group that consists of over 8000 healthy brains from 18 to 85 years, using the CE-certified volumetric tool AIRAscore (AIRAmed GmbH, Tübingen).

### CSF collection

Spinal tap was performed between 9.00 a.m. and 1.00 p.m. Samples were centrifuged within 60 and frozen at −80 °C within 90 min after collection. All samples have been analyzed in our clinical lab for the following routine diagnostic markers and only samples with normal routine levels were included, Table 2.

**Table 2:** Routine diagnostic CSF levels.

	Unit	Reference
CSF inspection	Clear	
Erythrocytes	Tausd/µl	<1
Cell count	1/µl	0–5
Leukocytes	1/µl	0–5
Polymorphonuclear cells	%	
Mononuclear cells	%	
Lactate in CSF	mmol/l	0–2.2
Albumin CSF/serum-quotient		×10E−3
IgG CSF/serum-quotient		×10E−3
Glucose CSF/serum-quotient		>0.5
Protein in CSF	mg/dl	0–45

### CSF α-Syn real-time quaking-induced conversion assay (RT-QuIC)

Purification of recombinant wildtype human α-Syn and the RT-QuIC assay were performed as described previously^35^. The same negative control and positive control samples were run throughout all experiments to optimize the comparison between fluorescent responses in different plates. To overcome batch-to-batch variations and intrinsic plate-to-plate variability, relative fluorescent units (RFU) were normalized for every time point in relation to the maximum intensity reached by the positive control within each plate and expressed as percentage. We calculated the threshold as the average normalized fluorescence value of negative control repeats during the first 10 h of recording, plus 30 standard deviations. The cut-off was set at 30 h. When only one of the four replicates crossed the threshold, the analysis was considered “unclear”. In those participants who showed a positive RT-QuIC α-Syn seeding profile (at least 2 out of 4 runs), we measured the area under the curve (AUC), the peak of the fluorescence response (Imax), and the lag phase (LAG) (time required to reach the threshold).

RT-QuIC experiments were performed at the Institute of Neurological Science of Bologna (ISNB). Results were reported blinded of the clinical diagnosis and genetic status. The assay previously showed a high specificity (98.7%) for Lewy-body pathology in a series of 121 CSF samples from individuals referred to the laboratory of neuropathology at ISNB for dementia of various etiologies in which the presence of Lewy-bodies and abnormal α-Syn deposits was excluded by neuropathological examination^35^.

Notably, the data of the RT-QuIC seeding profiles of the 107 PD_wildtype_, 99 PD_GBA_ and 26 healthy controls have been reported previously within another analysis^12^. We performed the RT-QuIC runs for the SNCA_Triplication_ patient exactly under the same experimental conditions and also used the same positive controls as in the previous analysis. Thereby, the normalization of the plate is fully comparable. Further, we also used the same threshold.

Detailed information on the RT-QuIC experiments are as follows:

#### Purification of human recombinant alpha-synuclein (α-Syn)

Glycerol stock of *E. coli* bacteria containing the vector for wild-type (wt) human α-Syn expression was obtained from Dr. Byron Caughey’s lab. The purification of the recombinant α-Syn was performed as reported^36^, with minor modifications: Bacteria from the glycerol stock were inoculated into 5 ml of Luria Broth (LB, Sigma) containing 50 µg/ml of kanamycin (Sigma) and let grow for 4–5 h at 37 °C with continuous agitation at 250 rpm. The initial culture was then added to 1 l of LB containing 50 µg/ml of kanamycin plus the overnight express auto-induction system (Merk-Millipore) in a full baffled flask. Cells were grown in a shaking incubator at 37 °C, 200 rpm overnight. The next day, the culture was split into four 250 ml flasks, and bacteria were harvested by centrifugation at 3200 × *g* for 10 min at 4 °C. The pellet was gently re-suspended in 25 ml osmotic shock buffer containing 40% sucrose, 2 mM EDTA, and 30 mM Tris at pH 7.2 using a 25 ml serological pipette and incubated 10 min at room temperature under mild agitation. Next, the suspension was centrifuged at 7900 × *g*, 20 m at 20 °C. The supernatant was discarded, and the pellet was re-suspended in 10 ml of ice-cold water for each pellet. Suspensions were pooled into two 50 ml tubes to a final volume of 20 ml per tube. 20 µl of saturated MgCl_2_ was added to each 20 ml suspension and incubated on ice for 3 min under mild rocking. Next, the suspension was centrifuged at 9000 × *g*, 30 min at 4 °C. The pellet was discarded, and the supernatant collected into a 100 ml glass beaker containing a magnetic stir bar. The pH was reduced to pH 3.5 by adding 400–600 µl HCl 1 M and incubated under stirring for 10 min at room temperature. Tubes were centrifuged at 9000 × *g* for 30 min at 4 °C, the pellet was discarded, and the supernatant collected into a fresh 100 ml glass beaker containing a magnetic stir bar. The pH was adjusted to 7.5 by adding 400–600 µl NaOH 1 M. The protein extract was filtered through a 0.22 µm filter (Merk-Millipore), loaded into a Ni–NTA column (Cytiva) on an NGC chromatographic system (Bio-Rad) and washed with 20 mM Tris, pH 7.5 at room temperature. The column was further washed with 50 mM imidazole in Tris 20 mM, pH 7.5, generating a peak that was not collected. A linear gradient up to 500 mM imidazole in 20 mM Tris, pH 7.5 was performed, and the peak was collected between 30 and 75% of imidazole buffer (150 and 375 mM, respectively). This peak was loaded onto an anion exchange column Q-HP (Cytiva) and washed in Tris 20 mM, pH 7.5, followed by another washing in 100 mM NaCl in Tris 20 mM, pH 7.5. A linear gradient up to 500 mM of NaCl in Tris 20 mM pH 7.5 was performed to collect the peak between 300 and 350 mM NaCl. The recovered fractions were pooled together and filtered through a 0.22 µm filter and dialyzed against water overnight at 4 °C using a 3.5 kDa MWCO dialysis membrane (Thermo-Scientific). The next day, the protein was moved into fresh water and dialyzed for four more hours. The protein concentration was measured with a spectrophotometer using a theoretical extinction coefficient at 280 nm of 0.36 (mg/ml)^−1^ cm^−1^. Finally, the protein was lyophilized using a lyophilizer (Thermo-Scientific) for 6 h and stored in aliquots at a final concentration of 1 mg/ml once re-suspended into 500 µl of hosphate buffer (PB) 40 mM, pH 8.0. Lyophilized aliquots were stored at −80 °C until usage.

#### The RT-QuIC assay

The RT-QuIC reactions were performed following an established protocol^36^. Black 96-well plates with a clear bottom (Nalgene Nunc International) were pre-loaded with six 0.8 mm silica beads (OPS Diagnostics) per well. CSF samples were thawed and vortexed 10 s before use. Fifteen µl of CSF were added as seed to trigger the reaction in 85 µl of buffer containing 40 mM PB, pH 8.0, 170 mM NaCl, 10 mM thioflavin-T (ThT), 0.0015% sodium dodecyl sulfate (SDS), and 0.1 g/l of recombinant α-Syn filtered using a 100 kDa MWCO filter (Pall-Life Sciences). The plate was sealed with a plate sealer film (Nalgene Nunc International) and incubated into Fluostar Omega (BMG Labtech) plate reader at 42 °C with intermittent double orbital shaking at 400 rpm for one minute, followed by 1-min rest. ThT fluorescence measurements were taken every 45 min using 450 nm excitation and 480 nm emission filter. To overcome batch-to-batch variations of α-Syn activity and the intrinsic experimental variability, we normalized the relative fluorescent units at each time point according to the fluorescence peak reached by the positive control and expressed the values as percentages. Intra-batch and inter-batch coefficients of variation (%) of quantitative RT-QuIC parameters Imax and AUC of the positive control after normalization were 6.8 and 11.8%, Table 3.

**Table 3:** Intra-batch and inter-batch coefficients of variation (%) of quantitative RT-QuIC parameters of the positive control, before (raw) and after normalization.

		Imax		AUC	
	α-Syn Batch no.	Norm CVs %	Raw CVs %	Norm CVs %	Raw CVs %
Positive control	1	4.7	9.2	10.4	14.9
	2	5.8	18.5	9.8	21.7
	3	7.2	20.1	7.7	26.9
	4	9.8	24.8	7.8	26.1
	5	6.0	22.1	16.8	32.5
	6	7.1	34.6	12.3	42.8
	7	2.5	37.1	19.2	52.2
	Overall CV %	6.8	22.3	11.8	26.0

The intra-batch coefficients of variation (CV) of the maximum intensity of fluorescence (Imax) and area under the curve (AUC) are expressed as percentage of the ratio between standard deviation and average.

Samples were run in quadruplicates and deemed positive when at least two out of four replicates reached the threshold, calculated as the average normalized fluorescence value of negative control repeats during the first 10 h of recording, plus 30 standard deviations. The analysis was repeated when only one replicate crossed this threshold.

#### Statistical analysis

RT-QuIC relative fluorescence responses were analyzed and plotted using the software Graphpad Prism 8.3. The AUC, the maximum intensity of fluorescence (Imax), and the lag phase were extracted.

#### CSF measurement of total α-Syn

CSF levels of total α-Syn were assessed using an ELISA kit for human α-Syn (Roboscreen GmbH, Germany). Intra-assay variation was 4.4% and calculated from duplicate analyses and expressed as median of the range to average of the duplicates. Inter-assay variation was determined using two quality control CSF pool samples and was <10%.

#### CSF measurement of Aβ_1-42_, total-Tau, phospho181-Tau, neurofilament light protein (NFL)

CSF levels of Aβ_1-42_, t-Tau and p181-Tau were measured blinded to the genetic status using ELISA kits from INNOTEST, Fujirebio GmbH, Germany. CSF levels of NFL were also measured blinded to the genetic status using the UmanDiagnostics NF-light®assay. Intra-assay coefficients of variation for each CSF parameter were below 15%.

### Skin biopsy and α-Syn staining

Skin punch biopsies were taken from the distal and proximal leg and fixed with paraformaldehyde and cryoconserved^37^. Twenty micrometer cryosections were cut. Double-immunofluorescence labeling was performed using anti-PGP9.5 (axonal marker, Zytomed Systems, Berlin, Germany, 1:200), anti-phospho-α-Syn (Biolegend, San Diego, CA, USA, 1:500) and appropriate Cy3- and AlexaFluor488-conjugated secondary antibodies (Jackson ImmunoResearch, West Grove, PA, USA, Invitrogen Fisher Scientific, Waltham, MA, USA/1:400/1:200). Double-immunofluorescence labeling was assessed blinded to diagnosis and genetic status using a fluorescence microscope with CARVII system (Ax10, Zeiss, Oberkochen, Germany/Visitron GmbH, Puchheim, Germany).

All slides were evaluated for phospho-α-Syn-positive dermal nerve fibers. Nerve fibers were identified by anti-PGP9.5 staining and only phospho-α-Syn deposition within nerve fibers was considered “positive.” Phospho-α-Syn-positive nerve fibers were categorized as sudomotor, vasomotor, pilomotor or somatosensory according to their location. Nerve fibers that could not be assigned to a certain skin structure were assessed as dermal nerve bundles. Phospho-α-Syn deposition was quantified as the number of skin structures that contained at least one phospho-α-Syn-positive nerve fiber. Importantly, skin biopsy data from 9 out of 10 previously reported PD_GBA_ (same collaboration between our group and the group of Dr. Doppler^38^) have been also included in the present manuscript along with CSF data on RT-QuIC α-Syn seeding profiles.

### Statistical analysis

As no published CSF data in patients with *SNCA* triplication are available, we descriptively compared clinical and CSF profiles of the SNCA_Triplication_ patient to PD_GBA_ as these are also specifically associated with prominent α-Syn pathology as well as to PD_wildtype_.

## Data Availability

Anonymized clinical data and biomaterial (plasma, serum, PBMC, fibroblasts and CSF) are available upon request to: kathrin.brockmann@uni-tuebingen.de.
